# Biofilms of the non-tuberculous *Mycobacterium chelonae* form an extracellular matrix and display distinct expression patterns

**DOI:** 10.1016/j.tcsw.2020.100043

**Published:** 2020-08-05

**Authors:** Perla Vega-Dominguez, Eliza Peterson, Min Pan, Alessandro Di Maio, Saumya Singh, Siva Umapathy, Deepak K. Saini, Nitin Baliga, Apoorva Bhatt

**Affiliations:** a School of Biosciences and Institute of Microbiology and Infection, University of Birmingham, Edgbaston, Birmingham B15 2TT, United Kingdom; b Institute for Systems Biology, Seattle, WA 98109, USA; c Department of Inorganic and Physical Chemistry, Indian Institute of Science, Bangalore 560012, India; d Department of Molecular Reproduction, Development and Genetics, Indian Institute of Science, Bangalore 560012, India

**Keywords:** NTMs, Non-tuberculous mycobacteria, ECM, Extracellular matrix, SEM, Scanning electron microscopy, DEG, Differentially expressed genes, eDNA, Extra cellular DNA, FMA, Free mycolic acids, TDM, Trehalose dimycolate, PG, Phosphatidyl glycerol, Non-tuberculous mycobacteria, *Mycobacterium chelonae*, Biofilms, Extracellular matrix, Raman spectroscopy, Lipids

## Abstract

*Mycobacterium chelonae* is an environmental, non-tuberculous mycobacterial species, capable of causing infections in humans. Biofilm formation is a key strategy used by *M. chelonae* in colonising niches in the environment and in the host. We studied a water-air interface (pellicle) biofilm of *M. chelonae* using a wide array of approaches to outline the molecular structure and composition of the biofilm. Scanning electron micrographs showed that *M. chelonae* biofilms produced an extracellular matrix. Using a combination of biochemical analysis, Raman spectroscopy, and fluorescence microscopy, we showed the matrix to consist of proteins, carbohydrates, lipids and eDNA. Glucose was the predominant sugar present in the biofilm matrix, and its relative abundance decreased in late (established) biofilms. RNA-seq analysis of the biofilms showed upregulation of genes involved in redox metabolism. Additionally, genes involved in mycolic acid, other lipid and glyoxylate metabolism were also upregulated in the early biofilms.

## 1 Introduction

Bacteria belonging to the genus *Mycobacterium* are predominantly environmental species, though some have evolved to become human and animal pathogens, including the causative agents of tuberculosis and leprosy (Bottai et al., 2014). A group of mycobacteria, termed non-tuberculous mycobacteria (NTMs), are capable of a dual lifestyle, usually occupying an environmental niche, but can cause a broad range of infections in humans (Falkinham, 2013). These include *Mycobacterium fortuitum*, and subspecies of the *Mycobacterium avium* complex (MAC) and *Mycobacterium abscessus* complex that cause pulmonary infections, and *Mycobacterium chelonae*, *Mycobacterium marinum* and *Mycobacterium ulcerans* that infect skin and soft tissue. In particular the members of the MAC are associated with HIV mortality (Corti and Palmero, 2008) and *M. abscessus* is often identified in the lungs of cystic fibrosis patients (Jönsson et al., 2007, Qvist et al., 2013). A key strategy for colonisation of both environmental and host niches by NTMs is the formation of biofilms (Falkinham, 2009). In the environment, NTM biofilms are found in water bodies including lakes, rivers and streams (De Groote et al., 2006, Iivanainen et al., 2010). The journey from the environment to host can also take place via intermediary or ‘man-made’ niches which includes tubing in hospital equipment, catheters, and plumbing for residential water supply (Falkinham, 2009). NTM biofilms in an infected host (Holland et al., 2017, Qvist et al., 2013, Qvist et al., 2015) are likely to play a key role in virulence (Faria et al., 2015), either by easing the colonization of the human host, evading the immune response, and/or fostering bacilli with an increased drug-tolerant phenotype (Aung et al., 2017, Aung et al., 2016, Davidson et al., 2011, Falkinham, 2009, Nessar et al., 2011, Orme and Ordway, 2014, Rhoades et al., 2019, Roux et al., 2016). Biofilms of NTMs can also act as reservoirs to seed bacteria into hosts (Benwill and Wallace, 2014, Gilbert, 2017, Jeon, 2019, Schreiber et al., 2018).

A number of mycobacterial species are known to forms biofilms in nature or *in vitro* (Chakraborty and Kumar, 2019, Zambrano and Kolter, 2005), and while some mycobacterial biofilm-associated phenotypes and components are common, other characteristics are species specific (Chakraborty and Kumar, 2019). An example of both can be found in the distinct lipids found in the mycobacterial cell envelope. Many mycobacterial biofilms accumulate free mycolic acids (FMA) (Ojha et al., 2010, Ojha et al., 2008, Sambandan et al., 2013) which form part of an extracellular matrix, and in *M. smegmatis* FMAs are released by enzymatic hydrolysis of trehalose dimycolate (TDM) by a cutinase-like serine esterase encoded by *MSMEG_1529* (Ojha et al., 2010). Both *M. smegmatis* and *M. tuberculosis* produce three subclasses of mycolic acids, of which the α-mycolates are found in both. Additionally *M. smegmatis* synthesises α’ and epoxy mycolates, while *M. tuberculosis* makes the oxygenated mycolates-methoxy and keto mycolic acids. Interestingly, keto mycolic acids, absent in *M. smegmatis* and other mycobacteria including NTMs, are also essential for biofilm formation in *M. tuberculosis* (Sambandan et al., 2013). Furthermore, glycopeptidolipids from the MAC and *M. smegmatis* play a key role in biofilm formation in these species (Freeman et al., 2006, Nessar et al., 2011, Recht and Kolter, 2001), but are not produced by other mycobacteria including members of the *Mycobacterium tuberculosis* complex.

*Mycobacterium chelonae* is an NTM that causes skin and soft tissue infections and is also the leading cause of mycobacterial ocular infections (Kheir et al., 2015). While *M. chelonae* has been often characterised as an opportunistic pathogen, causing infections in immunocompromised hosts who have undergone trauma or iatrogenic procedures, it has also been reported to infect individuals with no underlying immune deficiencies (Campbell et al., 2013, Jagadeesan et al., 2013). In this study we chose to study biofilms of *M. chelonae* for a number of reasons. First, *M. chelonae* is a rapid biofilm former and can form biofilms under a range of nutrient proficiencies (Hall-Stoodley et al., 1999). Next, across a range of niches, ranging from water bodies, medical equipment and catheters, to diverse infected regions in a host, including skin, cornea and implants, biofilm formation is a key strategy for *M. chelonae* colonisation (Falkinham, 2009, Martín-de-Hijas et al., 2009). Unlike a lot of other mycobacteria, *M. chelonae* has been shown to form biofilms *in vivo* (Aung et al., 2017, Chandra et al., 2001). And finally, *M. chelonae* is regarded as a highly drug tolerant NTM (Brown-Elliott et al., 2012, Cowman et al., 2016), attributed in part to the presence of strains with decreased expression of porins (Garcia et al., 2019, Svetlíková et al., 2009), as well as the presence of beta lactamases (Kwon et al., 1995) encoded in its genome (Fedrizzi et al., 2017), properties which potentially make it difficult to treat infections that involve biofilms of *M. chelonae*. Despite the importance of biofilms in the pathobiology of *M. chelonae*, we do not have a good understanding of how this NTM species forms biofilms, what they are composed of, and if they differ in composition to those of other mycobacteria.

Deciphering biofilm formation in *M. chelonae* not only sheds light on our understanding of mycobacterial biofilm formation in general, but also has implications for future therapies that combine strategies to weaken these structures in addition to standard antimicrobial regimes when treating NTM infections. In this study we aimed to study the molecular components of *M. chelonae* pellicles, a water-air interphase biofilm, using an array of approaches that use electron microscopy, confocal microscopy, Raman spectroscopy and other analytical methods to define its ultrastructure, and biochemical content. Furthermore, we used transcriptomics to outline distinct expression patterns in the pellicles compared to planktonic cultures, enabling us to conduct future studies outlining the temporal mechanisms of biofilm establishment and formation.

## 2 Materials and methods

### 2.1 Culture conditions

*M. chelonae* CCUG47445 was routinely grown in Middlebrook 7H9 (Difco^TM^) supplemented with OADC (Oleic acid-Albumin-Dextrose-Catalase, BD Difco^TM^) and 0.05% tyloxapol (Sigma-Aldrich) or 7H10 (Difco^TM^) supplemented with OADC (BD Difco^TM^) at 30 °C. For pellicle formation, logarithmic cultures (OD 0.8–1) were diluted in Sauton’s media supplemented with 0.5% glucose (Sigma), until an OD of 0.03, and growth in either on 24-well plates (for microscopy and lipid analysis) or on 75 cm^2^ cap-vented culture flasks (for transcriptomics and carbohydrate analysis), in a 30 °C static incubator for 5 (Biofilm t1) or 10 days (Biofilm t2). These specific time points were selected to capture two key transitions in *M. chelonae* biofilm formation, one occurring early (Biofilm t1) and linked to characteristic wrinkling of a mature mycobacterial pellicle. The second time point (Biofilm t2) aligned with a later event where the pellicular structure had sunk and was easily dispersed on agitation. For growing planktonic cultures, *M. chelonae* was inoculated in the same way as for pellicles, but tyloxapol was added to the cultures to a final concentration of 0.05%, and incubated at 100 rpm until an OD of 1.

### 2.2 RNA-Seq analysis

Biofilms (timepoint 1 and 2) and planktonic cultures from four different experiments were used for whole transcriptomics analysis. Total RNA was extracted from a 200 μL bacterial pellet, either from biofilms or planktonic bacteria. The bacterial pellets were resuspended in a lysis tube with 600 μL of a lysozyme (Amersham Pharmacia Biotec) solution (5 mg/mL in Tris-EDTA pH = 8, Thermo Fisher scientific) and 7 μL of β-mercaptoethanol (Sigma-Aldrich), and agitated at maximum speed in a FastPrep 120 Homogenizer (QBiogene) for one minute at room temperature. Following agitation, 60 μL of 10% sodium dodecyl sulfate (Sigma-Aldrich) were added to the mix, and the samples were homogenized at the same speed for two more minutes. To the recovered supernatant (600 μL), 60 μL of 3 M sodium acetate pH = 5.2 (Sigma-Aldrich) was added, followed by 720 μL of acid phenol pH = 4.2 (Fisher Bioreagents). After a five-minute incubation at 65 °C, the upper aqueous phase was recovered and washed once with 720 μL of acid phenol pH = 4.2, and once with 550 μL of chloroform/isoamyl alcohol 24:1 (Sigma-Aldrich). 400 μL of the recovered upper aqueous phase were mixed with 40 μL of 3 M sodium acetate pH = 5.2, followed by 3 volumes of chilled ethanol (Sigma-Aldrich). The RNA was precipitated overnight at 4 °C, and the obtained pellet was washed once with 70% ethanol. Once dry, the RNA pellet was resuspended in RNAse free water (Thermo Fisher Scientific), followed by a treatment with DNAse (Promega). The resulting RNA was quantified in the NanoDrop (Thermo Scientific), and its integrity was assessed in a 2100 Bioanalyzer system (Agilent Technologies).

The ribosomal RNA was depleted using the Ribo-Zero Gold rRNA Removal Kit (Illumina) according to the manufacturer directions. For synthesizing the DNA library, the Tru-Seq Stranded RNA (Illumina) and the samples were sequenced using an Illumina NextSeq Instrument. Paired-end 75 bp reads were checked for technical artifacts using Illumina default quality filtering steps. Raw FASTQ read data were processed using the R package DuffyNGS as described previously (Vignali et al., 2011). Briefly, raw reads were passed through a 3-stage alignment pipeline: (i) a prealignment stage to filter out unwanted transcripts, such as rRNA, mitochondrial RNA, albumin, and globin; (ii) a main genomic alignment stage against the genome(s) of interest. Reads were aligned to *M. chelonae* (ASM163280) with Bowtie2 (Langmead and Salzberg, 2012), using the command line option “very-sensitive.” No mitochondrial RNA, albumin, and globin genomes were provided for the bacterial samples. BAM files from stage (ii) was combined into read depth wiggle tracks that recorded both uniquely mapped and multiply mapped reads to each of the forward and reverse strands of the genome(s) at single-nucleotide resolution. Gene transcript abundance was then measured by summing total reads landing inside annotated gene boundaries, expressed as both RPKM and raw read counts. RNA-seq data (raw fastq files and read counts) have been deposited in the GEO repository under accession number GSE144514.

### 2.3 Differentially expressed genes

A panel of 5 differential expression (DE) analysis tools was used to identify gene expression changes between 5-day old biofilms (Biofilm t1) samples and planktonic samples or 10-day old biofilms (Biofilm t2) samples and planktonic samples. The tools included (i) RoundRobin (in-house); (ii) RankProduct (Breitling et al., 2004); (iii) significance analysis of microarrays (SAM) (Tusher et al., 2001); (iv) EdgeR (Robinson and Smyth, 2008); and (v) DESeq2 (Love et al., 2014). Each DE tool was called with appropriate default parameters and operated on the same set of transcription results, using RPKM abundance units for RoundRobin, RankProduct, and SAM and raw read count abundance units for DESeq2 and EdgeR. All 5 DE results were then synthesized, by combining gene DE rank positions across all 5 DE tools. Specifically, a gene’s rank position in all 5 results was averaged, using a generalized mean to the 1/2 power, to yield the gene’s final net rank position. Each DE tool’s explicit measurements of differential expression (fold change) and significance (*P*-value) were similarly combined via appropriate averaging (arithmetic and geometric mean, respectively). Genes with averaged absolute log2 fold change bigger than two and multiple hypothesis adjusted *P*-value below 0.01 were considered differentially expressed.

### 2.4 Analysis for metabolic pathway enrichment

We mapped the significantly differentially expressed genes at biofilm t1 and t2 against the most recent genome-scale metabolic network construction of *M. tuberculosis* H37Rv iEK1011 (Kavvas et al., 2018) by identifying orthologs using protein to protein sequence comparison using the BLOSUM62 scoring matrix (Henikoff and Henikoff, 1992). We used the subsystem definitions outlined in iEK1011 to explore pathway usage at the network level. We identified metabolic pathways that were significantly enriched in the *M. chelonae* biofilm stages (Benjamini Hochberg corrected hypergeometric *P*-value < 0.05). For these pathways, we calculated the average fold-change of all genes.

### 2.5 Raman spectroscopy

Raman spectra were collected from *M. chelonae* planktonic bacteria and biofilms (timepoint 1 and 2) using a Renishaw InVia Raman Microscope (Renishaw, UK) equipped with 785 nm laser. The laser was focussed onto the sample using a 50X objective with 0.75NA (Leica, Germany). Spectral calibration was performed using the 520.5 cm^−1^ Raman band for silicon. The laser power on the sample was 12 mW. Data collection was performed using the Wire 4.2 software with 8 s exposures and 10 accumulations.

The planktonic and biofilm bacterial cultures from each replicate (4 replicates) was pelleted (6000 X g, 4 °C, thrice) and resuspended in milliQ water. A concentrated bacterial solution of 2.5 µL was cast on MgF_2_ substrate (Global optics, UK). From each dried drop at least 30 spectra per experiment were collected from different areas of sample and each experiment was repeated thrice to account for biological heterogeneity.

Raman spectra were subjected to pre-processing steps. The spectra were checked for cosmic ray removal and baseline correction. All spectra were vector normalised to remove any effects related to concentration and instrumental variations using Origin 2016. To remove noise, the spectra were smoothened using 7 point, 3rd order polynomial -based analysis Savitzky- Golay smoothening using Wire 4.2. Multivariate analysis (PCA) were performed using Unscrambler X 10.3 (Camo Analytics, Norway).

To determine the main Raman shifts driving the variability between the samples, we applied a Principal Component Analysis in the normalized samples, using the SciKit-Learn (Pedregosa et al., 2011) module in Python, and we further associated the obtained Raman shifts with characteristic biomolecules as described before (Kuhar et al., 2018, Talari et al., 2015, Wiercigroch et al., 2017). The intensities between samples were compared using a Mann-Whitney u test, where the intensities of the samples were considered significantly different if the p-value < 0.05.

### 2.6 Scanning electron microscopy (SEM)

*M. chelonae* 5-day old biofilms (t1) were formed in a 24-well plate. 10-day old pellicles were easily disrupted due to movement, thus were not imaged. The formed biofilm was fixed overnight with a solution of 6% paraformaldehyde (Sigma Aldrich) in PBS, and imaged using a Philips XL-30 FEG ESEM in the Centre for Electron Microscopy at the University of Birmingham.

### 2.7 Confocal microscopy

eGFP-expressing *M. chelonae* biofilms t1 were formed as described for SEM, and stained with a single fluorophore targeting a specific component of the biofilm matrix. The conditions used for each fluorophore are summarized in Table 1. The stained pellicles then were fixed using paraformaldehyde 4% in PBS for 30 min, and mounted in microscope glass slides for further image acquisition. From three different experiments, five confocal z-stacks (covering approximately 4 μm) were acquired from each experimental sample. Images were acquired using a Nikon A1R system equipped with Ti microscope frame and a 100x/1.4 PlanApo objective.

**Table 1:** Fluorophores and conditions used for staining *M. chelonae* biofilms for CLSM.

Fluorophore	Target	Concentration	Time
Nile Red	Lipids	1 μM	30 min
Propidium iodide	Nucleic acids	15 μM	15 min
Sypro Ruby	Proteins	As provided by the manufacturer.	30 min
Alexa Fluor	α-mannose and α-glucose in the pyranose configuration.	100 μg/mL	30 min

### 2.8 Image processing

The acquired images (5 images per each experiment, 3 different experiments) were processed in Icy software (de Chaumont et al., 2012), using a similar approach as in Pike et al., 2017. Briefly, the acquired images were de-noised using a median filter, and for generating the region of interest, an automated threshold was calculated using the Li method (Li and Tam, 1998). Once the region of interest (ROI) was created, the Colocalization Studio plugin and the ROI Statistics plugins in Icy were used to calculate the Pearson’s and Mander’s coefficients, and the volumes of the matrix components respectively.

### 2.9 Lipid analysis

For lipid analysis, *M. chelonae* biofilms and planktonic cultures were grown as described before, but Sauton’s media was supplemented with ^14^[C]-acetic acid (1 μCi/mL, Perkin Elmer). Different lipid fractions were extracted and resolved by thin layer chromatography as described previously (Besra, 1998). Lipid species were visualised by autoradiography by exposing X-ray films Kodak Carestream) to the resolved TLC plates for 48 h.

### 2.10 Extraction and analysis of surface exposed carbohydrates

Surface exposed materials were extracted mechanically as described elsewhere (Grzegorzewicz and Jackson, 2013, Parish et al., 2003). The harvested pellets from three different experiments of planktonic cultures and biofilms were mixed with 4 mm glass beads and shaken gently for 2 min, and immediately after, the pellets were resuspended in 50 mL of miliQ water, and further centrifuged at 3000g for 15 min at 4 °C. The obtained supernatants were filtered through a 0.45 μm pore size filter, and concentrated to 1/10th of the original volume using a rotary evaporator (Buchi). The concentrated filtrate was mixed with chloroform and methanol to a final ratio chloroform/methanol/water 1:2:0.8 (v/v/v). The mix was agitated for 1 h, and then centrifuged for 10 min at 3000g. The aqueous phase and the interphase were recovered in separate tubes. The interphase was re-extracted three more times with miliQ water and the obtained supernatants were pooled with the previously recovered aqueous phase. The pooled extracts were concentrated to a final volume of 2 mL of miliQ water for further digestion with Proteinase K (Promega). The protein-digested material was dialyzed against MiliQ water for 48 h using a 3.5 kDa SpectralPore dialysis membrane (Spectrum Laboratories Inc.), and 10 μL of the obtained materials were hydrolysed with trifluoroacetic acid (Sigma-Aldrich) to obtain monosaccharides for further derivatization of alditol acetates for gas chromatography analysis as described previously (Grzegorzewicz and Jackson, 2013).

## 3 Results

### 3.1 Scanning electron microscopy (SEM) reveals the presence of a potential extracellular matrix (ECM) in M. Chelonae biofilms.

**Fig. 1:**
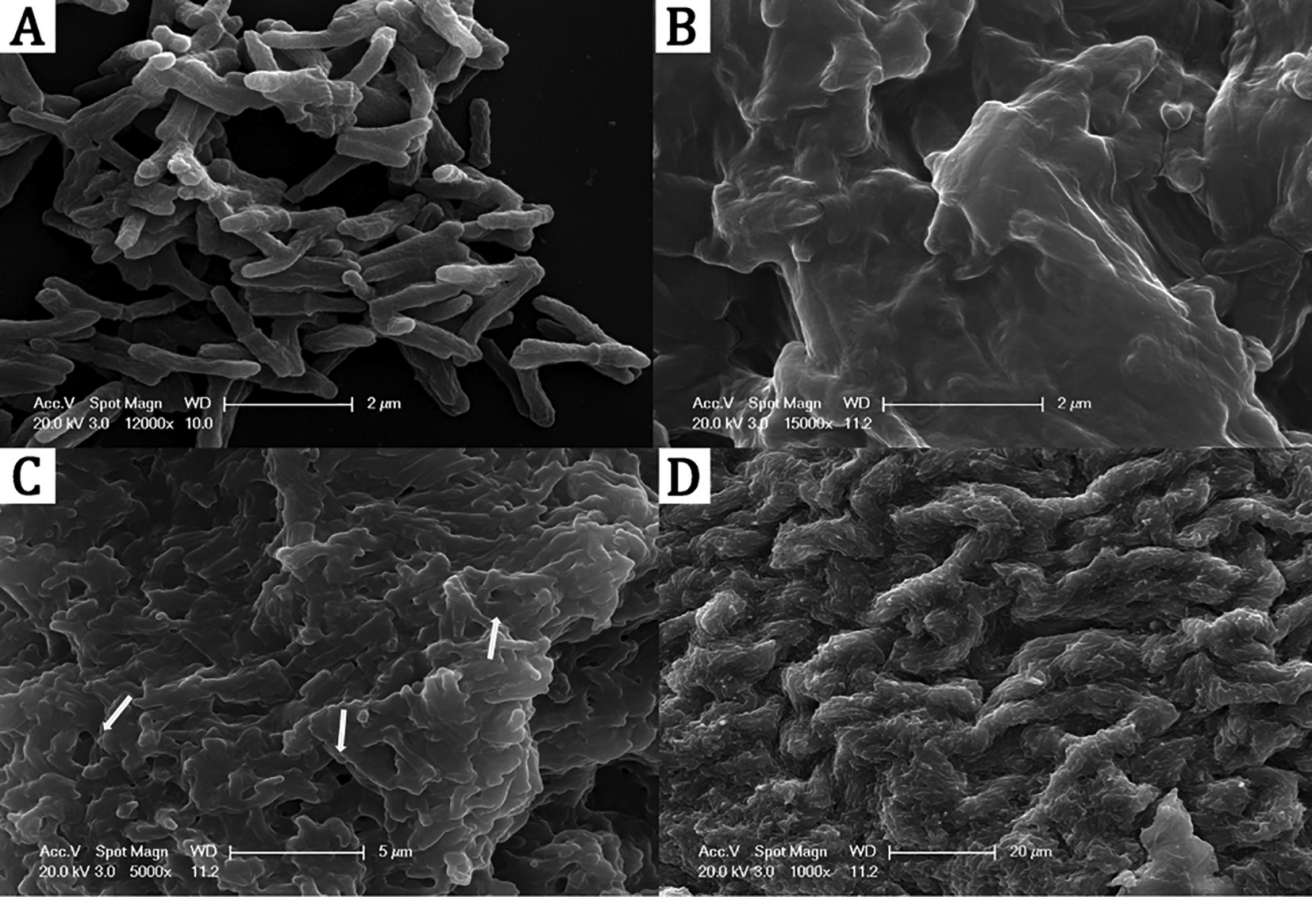
Scanning electron micrographs (SEMs) of *M. chelonae* cultures grown in Sauton’s media. Unlike planktonic *M. chelonae* where individual bacilli are clearly visible as rods (A), SEMs of 5-day old *M. chelonae* pellicles show the presence of contiguous mass of what appeared to be extracellular material (ECM) coating bacilli (B-D, decreasing magnification). The structures of the 5-day old *M. chelonae* pellicles showed the presence of pores (one such pore is highlighted with a white circle in C).

To visualise the detailed ultrastructure of *M. chelonae* biofilms, we first imaged a 5-day old biofilm (Biofilm t1) by SEM. The micrographs revealed the presence of a thick material covering mycobacterial growth, likely an extracellular matrix (ECM), with no clear outlines of individual bacterial cells within the pellicle (Fig. 1B–D). SEMs of planktonic cultures, on the other hand revealed individual mycobacterial cells (Fig. 1A), lacking any discernible extracellular material. The *M. chelonae* biofilm also revealed the presence of pores intermingled with cords of *M. chelonae*, suggesting a similar architecture to biofilms of other mycobacterial species (Fig. 1C–D) (Bardouniotis et al., 2001, Marsollier et al., 2007, Sambandan et al., 2013, Trivedi et al., 2016). Thus, the one remarkable characteristic revealed by SEM was the presence of a substantial ECM in the *M. chelonae* biofilm. The pores observed in the biofilm were likely conduits for nutrients to inner parts of the biofilm.

### 3.2 Raman spectroscopy reveals differing spectra for *M. chelonae* biofilms and planktonic cells

**Fig. 2:**
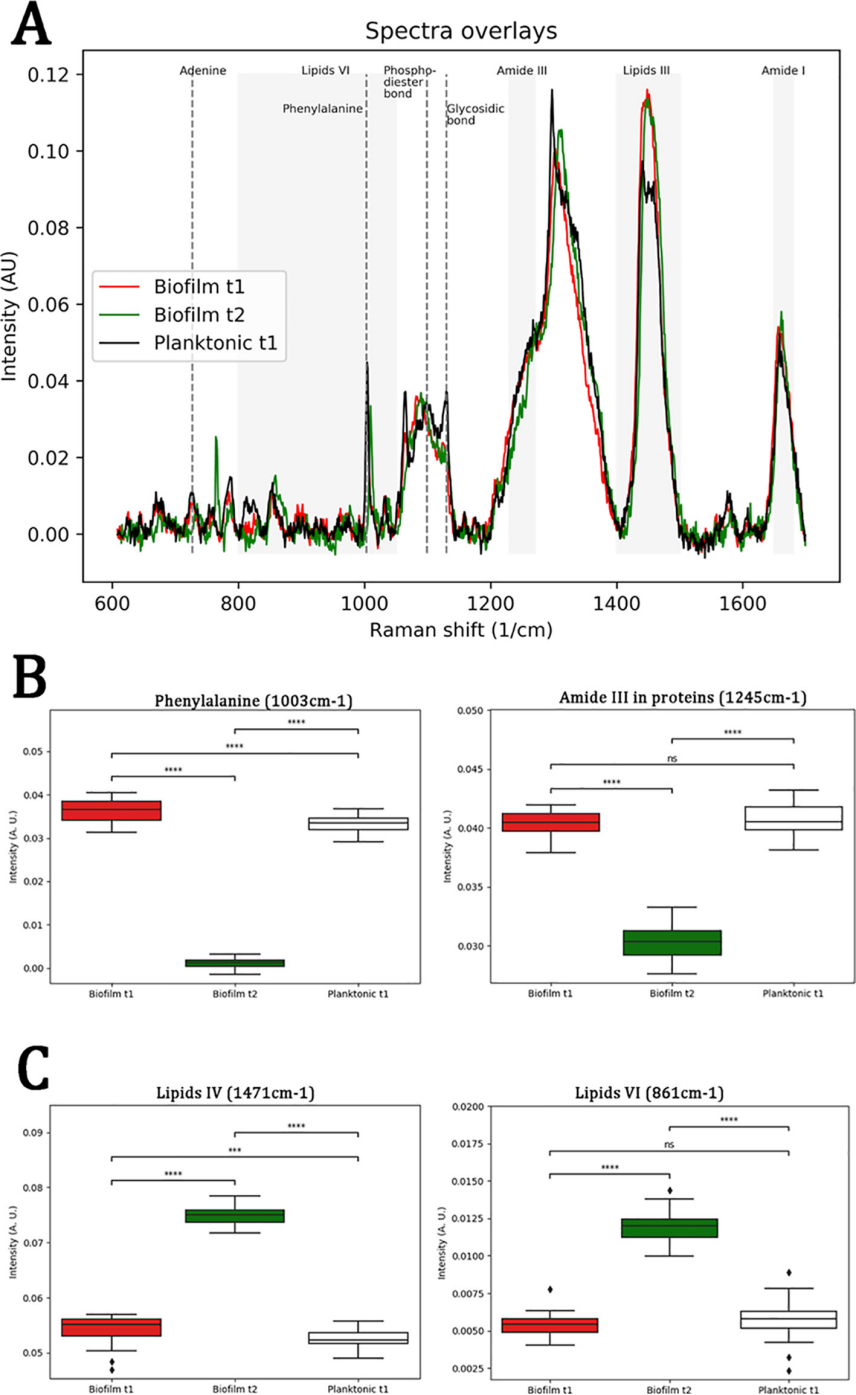
Raman spectra of *M. chelonae cultures*. Raman spectra was obtained from *M. chelonae* biofilms and planktonic cultures (Fig. 2A), and the principal component analysis (File S1, supplemental materials) show differences mainly in Raman shifts associated to proteins and lipids. A comparison of the medians of the intensities for characteristic Raman shifts associated with proteins (Fig. 2B; phenylalanine 1003 cm^−1^ and amide III 1245 cm^−1^) shows a uniform trend, where the medians of the intensity of the signals decrease for Biofilm t2 compared to Planktonic cultures or Biofilm t1; while the comparison of the intensities of the Raman shifts in regions associated with lipids (Fig. 2C; lipids region IV 1471 cm^−1^, and Lipids region VI, 864 cm^1^) are increased in Biofilm t2 compared to Biofilm t1 and Planktonic bacteria (C). The Raman shifts with high contribution to the principal components associated with nucleic acids (ring breathing of adenine, 726 cm^1^; phosphodiester bonds in DNA,788 cm^1^; symmetric stretching of PO_4_ in DNA, 1099 cm^−1^) and carbohydrates (glycosidic linkage, 1131 cm^−1^ and 941 cm^−1^) did not show a uniform trend (File S1, supplemental materials), likely due to the contribution of other biomolecules at the same wavelengths.

To further outline the biomolecular constituents of *M. chelonae* biofilms, we next queried whether biofilms had distinct biomolecule composition compared to planktonic cultures. We initiated these studies using Raman Spectroscopy (RS). We chose RS as it is a rapid approach to study the overall biochemical composition between biofilms and planktonic bacteria to outline differences between samples. Unlike other vibrational spectroscopic techniques, such as infrared spectroscopy, water does not cause interference, an attribute that makes Raman spectroscopy an attractive tool for studying intact biofilms with minimal processing requirements (Kelestemur et al., 2018). RS has been widely used to study bacterial biofilms (Kelestemur et al., 2018). It has also been used to study the biology of mycobacteria (Buijtels et al., 2008, Kumar et al., 2020, Perumal et al., 2018, Stöckel et al., 2017, Stöckel et al., 2015, Verma et al., 2019). Raman spectroscopy also has potential as a diagnostic tool, as it allows the identification of mycobacteria to the species level (Buijtels et al., 2008, Stöckel et al., 2017, Stöckel et al., 2015, Verma et al., 2019), and even to determine the viability of the identified bacilli (Kumar et al., 2020). We generated the Raman spectra from *M. chelonae* planktonic, as well as 5 day (Biofilm t1) and 10 day old biofilms (Biofilm t2) (Fig. 2A). While it was not possible to distinguish between samples to easily identify Raman peaks by overlaying the spectra, we were able to observe differences following Principal Component Analysis of the collected spectral data (File S1, supplemental materials). We found that 62.6% of the variance from the data set could be explained using three principal components (PC1 36.3%, PC2 23.6%, and PC3 2.7%), and we further compared the intensities of the signal of characteristics Raman shifts with high contribution to these PCs (File S1, supplemental materials). We then associated the identified Raman shifts with biomolecules using previously described Raman signatures (Kuhar et al., 2018, Talari et al., 2015, Wiercigroch et al., 2017). The analysis showed that the highest variability between *M. chelonae* planktonic growth and biofilms was for lipids (1400–1500 cm^−1^, lipids IV) and protein signals (1003 cm^−1^, phenylalanine; 1200–1300 cm^−1^, amide III), with a lesser variability for nucleic acids (726 cm^−1^, adenine; 791 cm^−1^, pyrimidine; 1099 cm^−1^, symmetric stretching of PO_4_^-^ in DNA) and carbohydrates (941 cm^−1^, α(1 → 6) glycosidic linkage; 1131 cm^−1^, symmetric stretching in glycosidic linkage) (Fig. 2A, File S1, supplemental materials). For proteins, we observed a decrease in the intensity of characteristics signals in Biofilm t2 (Fig. 2B), whereas the signals for lipids increased (Fig. 2C) in Biofilm t2.

### 3.3 Flourescence confocal microscopy of the *M. Chelonae* biofilm

**Fig. 3:**
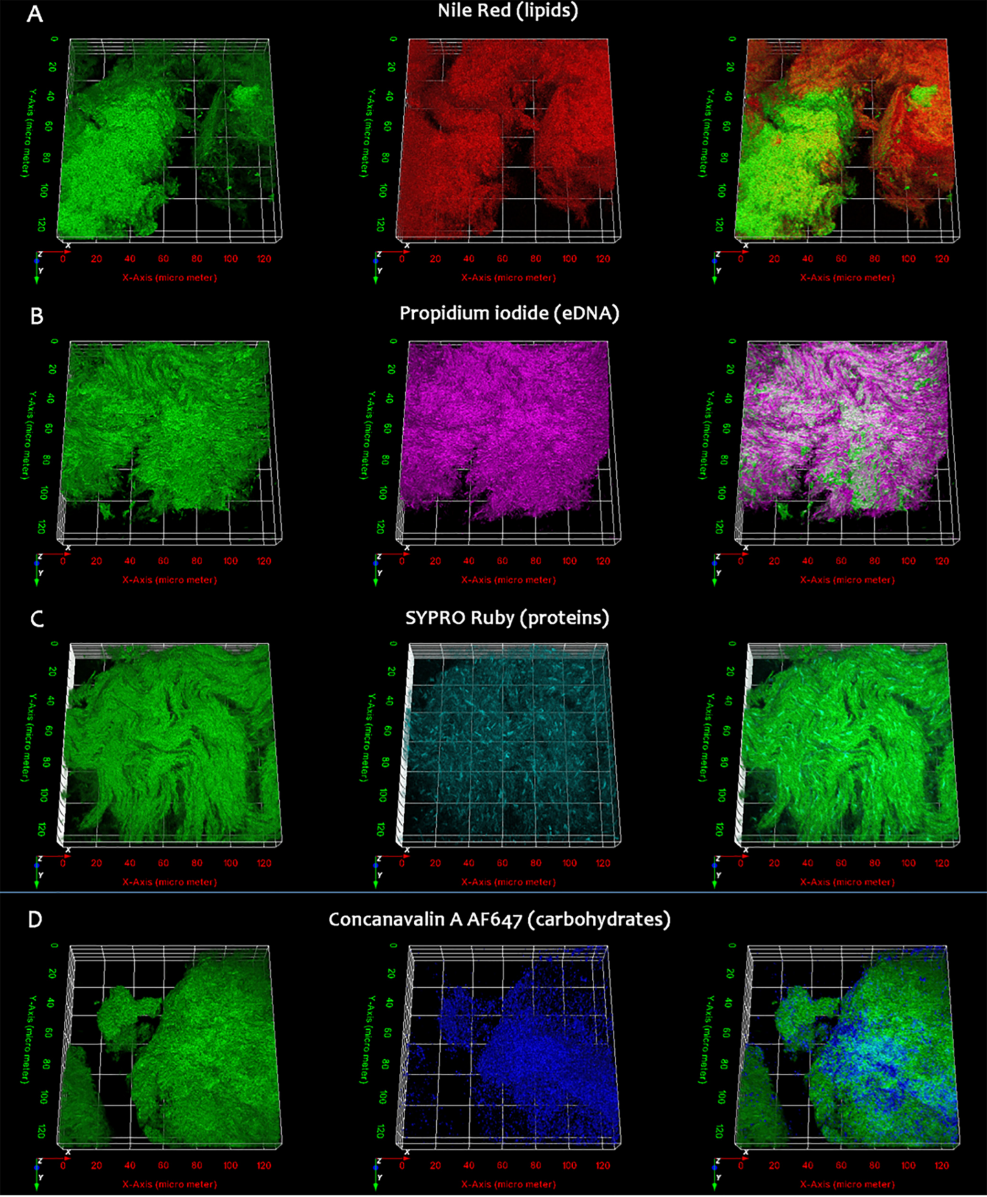
Confocal micrographs of 5-day old *M. chelonae* biofilms. 5-day old eGFP-expressing *M. chelonae* biofilms were stained separately with fluorophores targeting polymers from the biofilm matrix. Nile red (Fig. 3A) was used to stain lipids, Propidium Iodide (Fig. B) for eDNA, SYPRO Ruby biofilm stain (Fig, 3C) for proteins, and Concanavalin A conjugated with AlexaFluor 647 (Fig. 3D) for carbohydrates. From left to right, 3D projections confocal z-stacks for eGFP, the fluorophore targeting a component from the extracellular matrix, and the overlay of both signals.

To follow up on our findings of a potential ECM structure revealed by SEM of *M. chelonae* biofilms, and its biomolecular composition by RS analysis, we further studied the composition and architecture of the *M. chelonae* biofilms using confocal microscopy. While 5-day old biofilms (Biofilm t1) of eGFP-expressing *M. chelonae* were stained with an array of fluorophores to selectively label the components of the biofilm; 10-day old biofilms (Biofilm t2) were easily disrupted with the washes involved in staining, so imaging was not pursued. We used Nile Red (NR, Sigma Aldrich) for staining lipids, Concanavalin A conjugated with Alexa Fluor 647 (ConcA, Thermo Fisher Scientific) for staining carbohydrate (polysaccharide), FilmTracer™ SYPRO® Ruby biofilm matrix stain (SR, Thermo Fisher Scientific) for proteins, and Propidium Iodide (PI, Sigma Aldrich) for nucleic acids. With the exception of NR, that is a lipophilic molecule able to penetrate mycobacterial cell wall (Xu et al., 2014, Yu et al., 2012), and thus able to stain extracellular and intracellular lipids, *M. chelonae* is impermeable to all other dyes used for staining. As an example, we used PI to stain eDNA, as this dye is commonly used as a cell viability marker, because it can’t penetrate intact cell membranes. Confocal imaging of the biofilms revealed staining by all four fluorophores indicating the presence of lipids, proteins, carbohydrates and DNA at an extracellular location (Fig. 3). Remarkably, the distribution of some of these biomolecules was not uniform in the biofilm, with some showing ‘sectoring’ with areas of high levels, to sectors where no signal was detected. File S2, supplemental materials, shows binarized images of a confocal stack of eGFP expressing *M. chelonae* biofilms stained for lipids, carbohydrates, protein, and eDNA, visualised traversing from the bottom to the top of a 20 μm section of the biofilm. To quantify this observation, we then evaluated colocalization between the bacilli and each of the four biopolymers. These measurements helped us to objectively describe the biofilm matrix (Schlafer and Meyer, 2017). To quantitate colocalization, we calculated two sets of values for the fluorescence signals obtained: Pearson’s correlation coefficient and Mander’s coefficients. Pearson’s correlation coefficient allows us to assess how well two signals linearly correlate to each other. The higher the value of the Pearson’s correlation coefficient is, the more likely the intensity of one signal will linearly increase, proportionally to the other signal it is being compared to. For example, if the intensity of the fluorescence signal of one of the biomolecules is compared to that of bacterial cell expressed GFP, and both signals increase or decrease proportionally, there is a correlation of the signal with GFP will be indicated by a high Pearson’s co-efficient. However, if the intensity of the signal from a biomolecule does not change regardless of the amount of bacteria (eGFP signal), the resultant value of the Pearson coefficient is lower. Manders co-efficient on the other hand is a measure of the co-occurrence of two signals, measuring the fraction of a given signal that overlaps with a second signal. If the fluorescence signal of a labelled biomolecule completely overlaps with the bacterial GFP signal, the Manders co-efficient will be 1, while a Mander’s coefficient of 0 means that none of the two signals overlap, i.e. the biomolecule and the bacteria are in distinct, exclusive sectors. We also calculated the relative volume of each of the assessed biopolymer, using as a reference the volume of *M. chelonae* (eGFP) in the biofilm. All the coefficients are summarized in Table 2. The data indicated that while both eDNA and lipids showed a high level of colocalization with bacteria in the images (Fig. 3), lipids with their relatively lower calculated Mander’s coefficients were more scattered across the biofilm matrix than eDNA. Proteins colocalize well with the bacteria, however, only around the 70% of the signal from proteins overlap with the bacteria (Manders coefficient M2, Table 2), showing that proteins form the bulk component of the biofilm matrix. Finally, the samples stained for carbohydrates showed the lowest Pearson’s coefficient, meaning a weak linear correlation of the intensity of the signals, even though around 90% of the carbohydrate signal overlaps with the signal for the bacilli. This indicated that there are zones in the biofilm that accumulate larger amounts of carbohydrates, mostly in the matrix, compared to the carbohydrates occurring in the close proximity of the bacteria.

**Table 2:** Colocalization coefficients for *M. chelonae* biofilm components.

Component of the ECM	Relative volume	Pearson’s correlation coefficient	Mander’s coefficient	
			M1	M2
Nile Red (Lipids)	1.030	0.717	0.926	0.898
Propidium iodide (eDNA)	1.081	0.859	0.984	0.924
Concanavalin A Alexa Fluor 647 (Carbohydrates)	0.688	0.271	0.640	0.966
SYPRO Ruby (Proteins)	1.073	0.678	0.832	0.732

### 3.4 Pellicles of *M. chelonae* exhibit a different lipid profile compared to planktonic cells

**Fig. 4:**
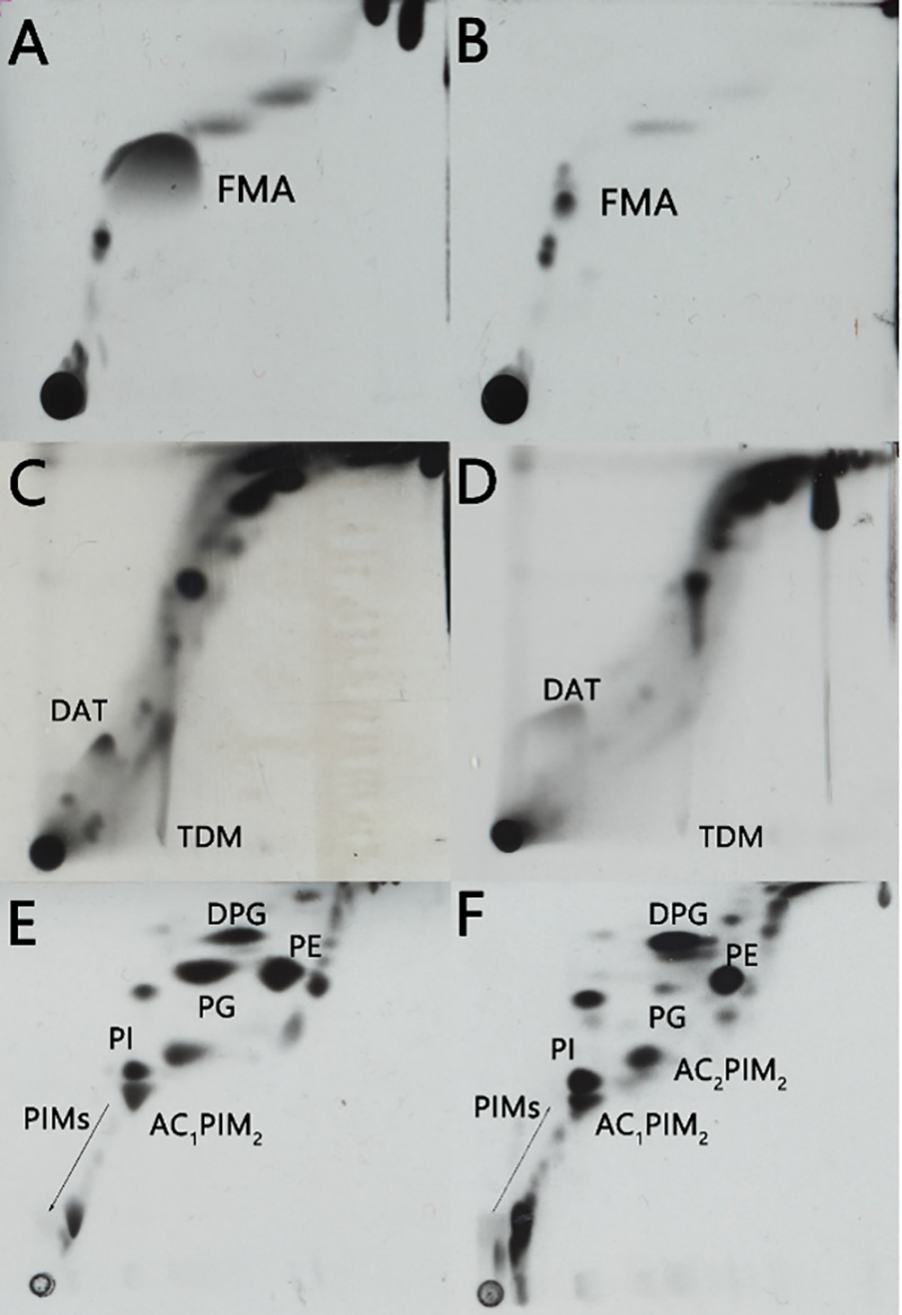
Lipid profile of *M. chelonae* cultures. Solvent extractable lipid fractions from *M. chelonae* biofilms and planktonic bacteria were resolved using thin layer chromatography, using solvent systems of different polarities. *M. chleonae* biofilms (Fig. 4A) show an accumulation of free mycolic acids (FMA) compared to planktonic cultures (Fig. 4B). The amount of trehalose dimycolate (TDM) decreases from biofilm T1 (5 days, Fig. 4C) to biofilm T2 (10 days, Fig. 4D). Polar lipids from *M. chelonae* biofilm T1 (Fig. 4E) show an accumulation of phosphatidyl glycerol (PG) compared to its planktonic counterpart (Fig. 4F). DAT- diacylthrealose, DPG- diphosphatidyl glycerol, PE- phosphatidyl ethanolamine, PI- phosphatidylinositol, AC_2_PIM_2_- diacetylated phosphatidylinositol dimannoside, AC_1_PIM_2_- acetylated phosphatidylinositol dimannoside, PIMs- phosphatidylinositol mannosides.

As mycobacteria produce a range of distinctive lipids, and given that several genes related to lipid metabolism are known to play a key role during mycobacterial biofilm formation, we chose to follow up on our microscopy and RS studies, by first looking at the lipid profiles of *M. chelonae* biofilms. Biofilm or planktonic *M. chelonae* cultures, were grown in Sauton’s media supplemented with ^14^C-acetic acid, and the extracted lipid fractions were resolved by thin layer chromatography, and visualized by auto-radiography (Fig. 4). Free mycolic acids were produced in excess by *M. chelonae* biofilms (Fig. 4A) when compared to those from planktonic cultures (Fig. 4B). Also, trehalose dimycolate (TDM) content decreased from biofilm t1 to biofilm t2 (Fig. 4C and 4D). Both lipid alterations have also been seen in other mycobacterial biofilms (Ojha et al., 2010). However, an alteration in a third class of lipids appeared distinct to *M. chelonae* biofilms: the amount of phosphatidylglycerol (PG) seems to be increased in *M. chelonae* biofilms (Fig. 4E) as compared to its planktonic counterpart (Fig. 4F), although this lipid is known to be scarce in the mycobacterial inner membrane (Jackson et al., 2000).

### 3.5 The glucose content of *M. chelonae* biofilms decreases as the biofilm ages

**Fig. 5:**
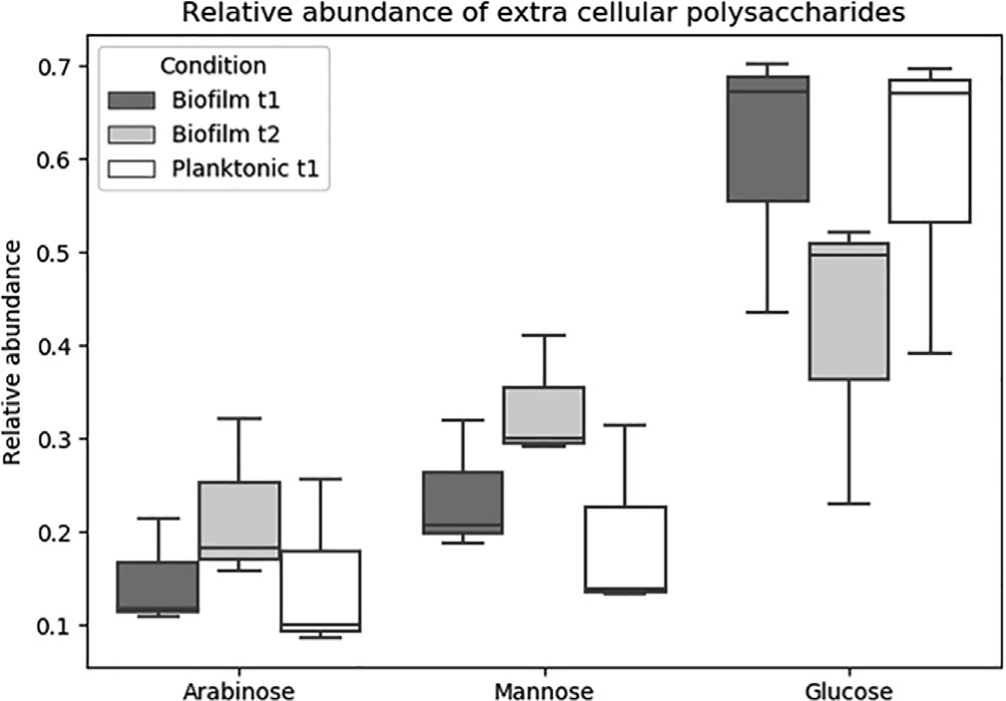
Relative abundance of sugars from extracellular polysaccharides. Polysaccharydes from the ECM of M. chelonae biofilms the three most abundant monosaccharydes present in *M. chelonae* biofilms and planktonic extracellular material are, in decreasing order, glucose, mannose, and arabinose. The relative abundance of glucose drops in Biofilm t2 compared to Biofilm t1 or planktonic bacteria.

To further study the nature of the carbohydrates detected in *M. chelonae* biofilm ECM by confocal microscopy and RS analysis, we assessed the composition of the polysaccharides present in the ECM. We separated the ECM from biofilm structure, and further purified polysaccharides by mechanically separating the ECM with glass beads, followed by chemical partition and protein digestion, prior dialyzing of the obtained aqueous phase using a 3 kDa membrane to remove salts and other small molecules (from media components). The purified extracts were hydrolysed with TFA to yield monosaccharides, which were in turn derivatized to alditol acetates, and resolved using gas chromatography (GC). The relative abundance of the principal monosaccharide components in the ECM of *M. chelonae* pellicles is summarized in Fig. 5. We found that the components of extracellular polysaccharides of *M. chelonae* biofilms, both t1 and t2, and planktonic cultures are glucose, mannose and arabinose; however, glucose stood out as being the most abundant. Interestingly, the proportion of the glucose content in the ECM polysaccharides decreases from biofilm t1 to biofilm t2.

### 3.6 *M. chelonae* biofilms display a distinct transcriptional profile

**Fig. 6:**
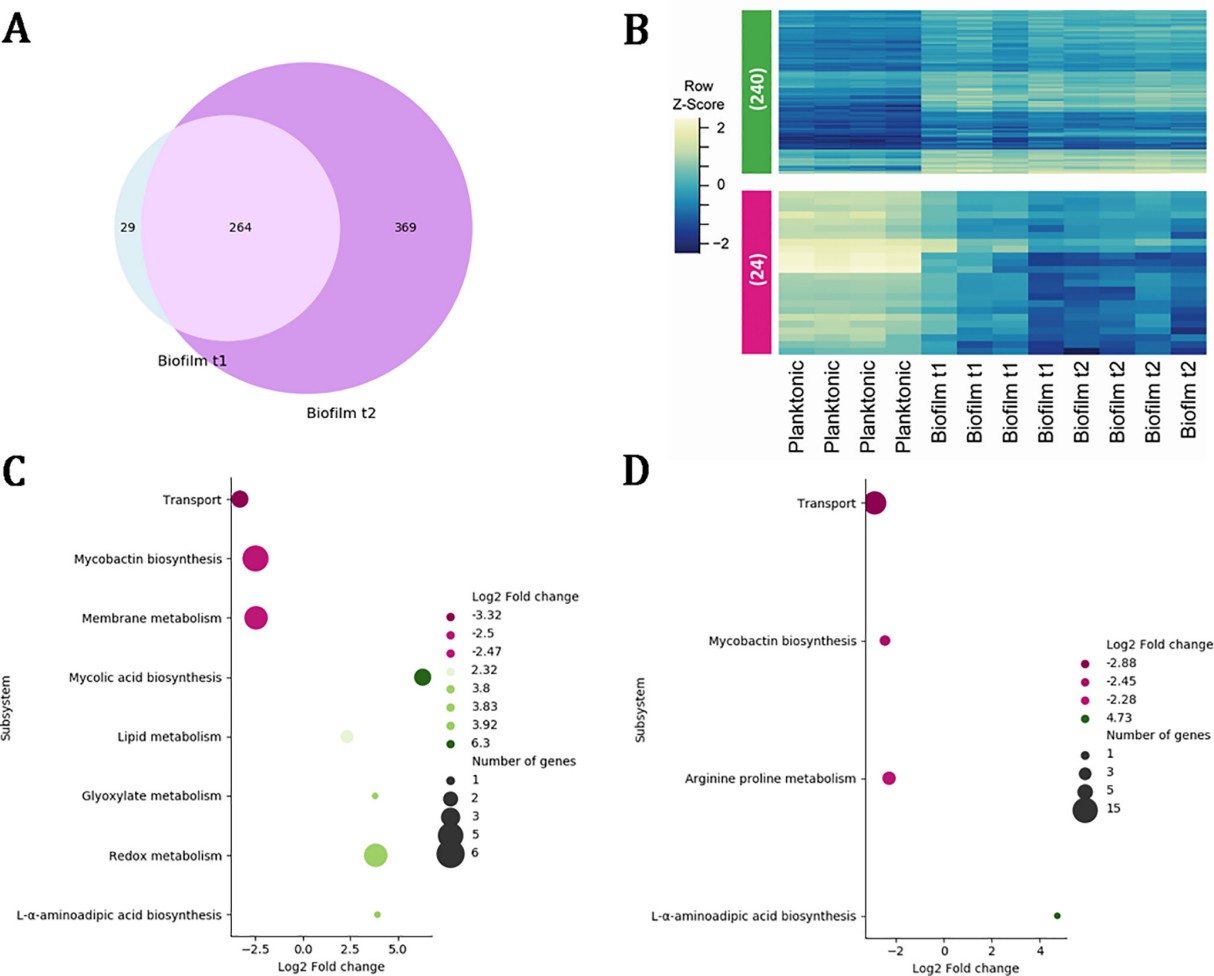
Differentially expressed genes and enriched metabolic subsystems in *M. chelonae* biofilms. The Venn diagram for DEGs (Fig. 6A) shows that 293 genes are significantly differentially expressed during Biofilm t1, while 633 are differentially expressed in Biofilm t2. There is a common set of 264 genes that show significant differential expression with the same directionality, and the raw Z-scores of the 240 up-regulated genes (Fig. 6B, green), and the 24 down-regulated genes (Fig. 6B, pink), are shown in the heatmap for biofilm T1 and T2 (Fig. 6B), suggesting a core group of genes required for the maintenance of the biofilm. The list of the enriched metabolic pathways, and their respective fold change, during Biofilm t1 and Biofilm t2 (Fig. 6C and Fig. 6D) shows that seven metabolic pathways are enriched during Biofilm t1, while only four are enriched during Biofilm t2. The genes that are differentially expressed on those metabolic pathways can be found in File S4 in supplemental materials.

To outline potential molecular mechanisms driving *M. chelonae* biofilm formation, we performed a transcriptomic analysis (RNA-seq) of *M. chleonae* biofilm t1 and t2, and compared these to that of a planktonic culture. Over all 293 genes were significantly differentially expressed (*P*-value < 0.01 and estimated absolute log2 fold-change > 2) in 5-day old biofilms (Biofilm t1), and 633 in 10-day old biofilms (Biofilm t2) (Fig. 6A). Identities of the DEGs are shown in File S3, supplemental materials. A total of 264 of these genes show significant differential expression with same directionality (i.e. up- or down-regulation) in both stages (Fig. 6B). The change in the expression of this set of genes could be due to the bacilli entering into the stationary phase, or perhaps because these genes have a role in biofilm maintenance. To depict an example, among the common genes differentially expressed with the same directionality in both biofilm stages, we found genes from the *mce5* operon, and *mce1A*. In *M. tuberculosis* the genes from the *mce* operons are upregulated during the stationary phase (Saini et al., 2008, Singh et al., 2016). In the context of biofilm, the deletion of all six of *mce* operons in *M. smegmatis* impairs the formation of this structure, likely due to alterations on the cell wall composition (Klepp et al., 2012). In this study, we found that genes of the *mce5* operon (*BB28_RS04495*/*yrbE5A*, *BB28_RS04475*/*mce5C*, *BB28_RS04470*/*mce5D*, and *BB28_RS04485*/*mce5A*) are downregulated during both biofilm stages, in contrast to what has been observed during the stationary phase in *M. tuberculosis.* We also observe an upregulation of the *mce1A* gene in both biofilm stages. The *mce1* operon aids *M. tuberculosis* to transition better from a slow growth rate state to a fast growth rate state (Beste et al., 2009), a trait that may result beneficial for the bacilli residing in a biofilm. These two examples suggest that, although some transcriptional changes occurring in the stationary phase are common to biofilm formation (upregulation of the *mce1A* gene), there are specific transcriptional signatures (downregulation of the *mce5* operon genes) occurring during biofilm formation.

To further query the metabolic pathways enriched during biofilm formation, we used a recently updated genome-scale model of *Mycobacterium tuberculosis* metabolism, iEK1011 (Kavvas et al., 2018), and looked for orthologs within the significantly differentially expressed genes from our transcriptional data. Eight metabolic pathways showed enrichment (Benjamini-Hochberg adjusted *P*-value < 0.05) in biofilm t1 (Fig. 6C), and four in biofilm t2 (Fig. 6D). The identities of the genes from each metabolic subsystem are summarized in supplemental materials (File S4). Transport genes and those for mycobactin biosynthesis are down-regulated in both biofilm t1 and t2. In addition, during biofilm t2 genes involved in the arginine and proline metabolism were down-regulated. Mycolic acid biosynthesis and other lipid metabolism were up-regulated during biofilm t1, as well as genes from the redox metabolism and glyoxylate pathway. Interestingly, *lat* (*BB28_RS18260*), coding for a lysine amino transferase (Tripathi and Ramachandran, 2006), is up-regulated in biofilm t1 and t2. In *M. smegmatis*, *lat* is involved in persister cell formation following exposure to norfloxacin (Li et al., 2016).

## 4 Discussion

*Mycobacterium chelonae*, like other clinically relevant NTMs, forms biofilms both in the environment and in the host. We have characterized *M. chelonae* pellicles, an *in vitro* biofilm model, describing the presence and composition of an ECM in the biofilm. We also delineated distinct transcriptional responses with potential roles in biofilm formation. The mechanisms involved in the development of mycobacterial biofilms are orchestrated as a response of fluctuations of redox state of the bacilli (Geier et al., 2008, Gupta et al., 2015, Koliwer-brandl et al., 2016, Ojha and Hatfull, 2007, Trivedi et al., 2016, Van De et al., 2016, Wolff et al., 2015), which in turn are generated due to the microenvironments within the biofilm. We observe an up-regulation of redox metabolism genes during Biofilm t1, specifically of subunits of the BD cytochrome, used in the electron transfer chain during hypoxic conditions. In *M. smegmatis* biofilms the NADH/NAD^+^ ratio is three times higher than in planktonic *M. smegmatis* (Anand et al., 2015), suggesting a reductive environment in bacilli within biofilms. Mycobacteria can use a variant of the TCA cycle that reduces oxaloacetate to succinyl CoA to replenish the NAD^+^ pool (Beste et al., 2011), thus helping to maintain the redox homeostasis in mycobacteria. During Biofilm t1, we observe an up-regulation of the lipid, glyoxylate, and mycolic acid metabolism. It would be interesting to measure metabolites associated with the redox state of the bacilli during *M. chelonae* biofilms, and also query the metabolic pathways active during biofilm formation/dispersion, to further explore potential anti-biofilm strategies.

Recently the molecular events defining the stages during pellicle formation in *Mycobacterium smegmatis* have been defined (Yang et al., 2017), and the role of lipid metabolism during biofilm formation has been extensively addressed in several mycobacteria (Anand et al., 2015, Nessar et al., 2011, Ojha et al., 2005, Ojha et al., 2010, Ojha et al., 2008, Pacheco et al., 2013, Pang et al., 2012, Recht and Kolter, 2001, Zambrano and Kolter, 2005). Our data shows that similar to other mycobacteria (Ojha et al., 2008, Ojha et al., 2010), *M. chelonae* biofilms accumulate free mycolic acids, likely from trehalose dimycolate (TDM), suggesting a similar mechanism as in *M. smegmatis*, where a serine-hydrolase cleaves TDM to yield free mycolic acids (Ojha et al., 2010). Interestingly, the accumulation of free mycolic acids has also been observed in a *M. tuberculosis* strain lacking Mez, an enzyme involved in the conversion of malate into pyruvate (Basu et al., 2018). Interestingly, biofilms of *M. chelonae* seem to accumulate phosphatidyl glycerol (PG), an inner membrane polar lipid species present, but not relatively abundant, in planktonic cells.

Our confocal microscopy analysis shows that proteins, and in a minor proportion, carbohydrates, are present in the biofilm matrix. Previous studies have shown that the most abundant component of *M. smegmatis* and *M. phlei* pellicles is proteins, and to a lesser degree, carbohydrates (Lemassu et al., 1996b) suggesting similarities between the biofilms of these mycobacterial species, and contrasts with *M. tuberculosis* (Ortalo-Magne et al., 1995) and *M. avium* (Lemassu et al., 1996a) biofilms, where the major component is carbohydrates. The composition of the extracted polysaccharide was predominantly glucose suggesting a cellulose or α-glucan polymer, followed by mannose and arabinose, likely from mannans and arabinomannans. Following extraction of exposed polysaccharides from *M. chelonae* biofilms, we noted a decrease in the glucose content from biofilm t1 to biofilm t2, which is likely to coincide with the dispersal stage of the biofilm. Other biofilm-forming bacteria are known to modulate polysaccharide metabolism as a strategy for biofilm dispersion (McDougald et al., 2012), as it is a crucial structural component of biofilms (Rathinam et al., 2019, Sutherland, 2001). In *Pseudomonas aeruginosa*, the quorum sensing molecule, N-acylhomoserine lactone, induces a signalling cascade that represses the *pel* operon and subsequently the synthesis of the Pel polysaccharide, which is abundant in *P. aeruginosa* biofilms (Ueda and Wood, 2009). Although quorum sensing molecules have not yet been identified in mycobacteria, it is possible that other signals pertaining to the later stages of the biofilm trigger mechanisms that lead to the repression of the synthesis of biofilm components, such as polysaccharides.

Along with lipids, carbohydrates and proteins, extracellular DNA (eDNA) is present in the matrix of several mycobacterial biofilms (Ackart et al., 2014, Rose et al., 2015, Rose and Bermudez, 2016, Trivedi et al., 2016). eDNA mediates the adhesion of bacteria to substrates prior to biofilm formation, and plays a role in the structural maintenance and protection against antimicrobials in several bacterial pathogens (Okshevsky and Meyer, 2015). *M. chelonae* forms biofilms with abundant eDNA in a keratitis murine model (Aung et al., 2017), and in mycobacteria, eDNA degradation increases the killing effect of some antibiotics, both *in vitro* and *in vivo* (Ackart et al., 2014, Aung et al., 2017, Aung et al., 2016, Rose et al., 2015). The pellicles formed by *M. chelonae* accumulate a significant amount of eDNA, suggesting that this type of biofilm could be used to resemble *in vivo* biofilms for further studies.

A thorough understanding of clinically relevant mycobacterial biofilms, such as *M. chelonae*, could contribute to a better understanding about the key components in NTM biofilms required for colonizing different environments within the human host, and would also contribute to a more rational design of therapeutics against NTM infections driven by biofilms. Our studies highlight the utility of Raman Spectroscopy and fluorescence, confocal microscopy to study the architecture and composition of *M. chelonae* biofilms. Additionally, the outlining of distinct gene expression patterns in *M. chelonae* pellicles enables us to conduct further studies on the mechanisms of *M. chelonae* biofilm formation.

## CRediT authorship contribution statement

**Perla Vega-Dominguez:** Conceptualization, Investigation, Visualization, Validation, Methodology, Formal analysis, Writing - original draft, Writing - review & editing. **Eliza Peterson:** Conceptualization, Investigation, Visualization, Validation, Methodology, Formal analysis, Writing - original draft, Writing - review & editing. **Min Pan:** Investigation, Validation, Methodology. **Alessandro Di Maio:** Methodology, Formal analysis, Writing - review & editing. **Saumya Singh:** Investigation, Methodology, Formal analysis, Writing - review & editing. **Siva Umapathy:** Conceptualization, Writing - review & editing. **Deepak K. Saini:** Conceptualization, Writing - review & editing. **Nitin Baliga:** Conceptualization, Writing - review & editing. **Apoorva Bhatt:** Conceptualization, Writing - original draft, Writing - review & editing.

## Declaration of Competing Interest

The authors declare that they have no known competing financial interests or personal relationships that could have appeared to influence the work reported in this paper.
