# Supplementary material for: Biofilms of the non-tuberculous Mycobacterium chelonae form an extracellular matrix and display distinct expression patterns
Source: Cell Surf. 2020 Aug 5;6:100043. doi: 10.1016/j.tcsw.2020.100043 (PMC7421604; doi:10.1016/j.tcsw.2020.100043)
Supplement: Supplementary data 2 [file mmc2.pptx]

## Slide 1
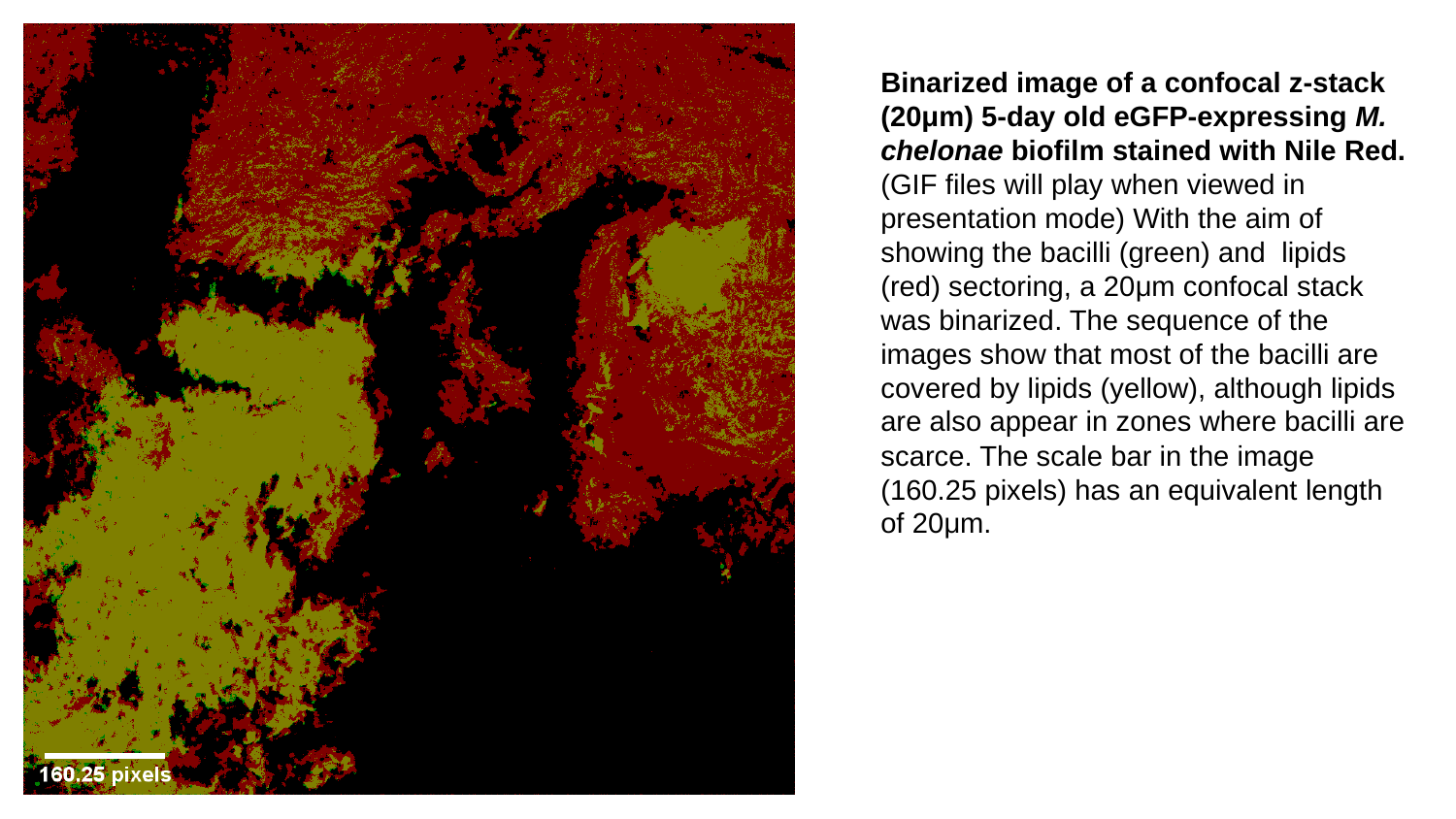

Binarized image of a confocal z-stack (20μm) 5-day old eGFP-expressing M. chelonae biofilm stained with Nile Red. (GIF files will play when viewed in presentation mode) With the aim of showing the bacilli (green) and lipids (red) sectoring, a 20μm confocal stack was binarized. The sequence of the images show that most of the bacilli are covered by lipids (yellow), although lipids are also appear in zones where bacilli are scarce. The scale bar in the image (160.25 pixels) has an equivalent length of 20μm.

## Slide 2
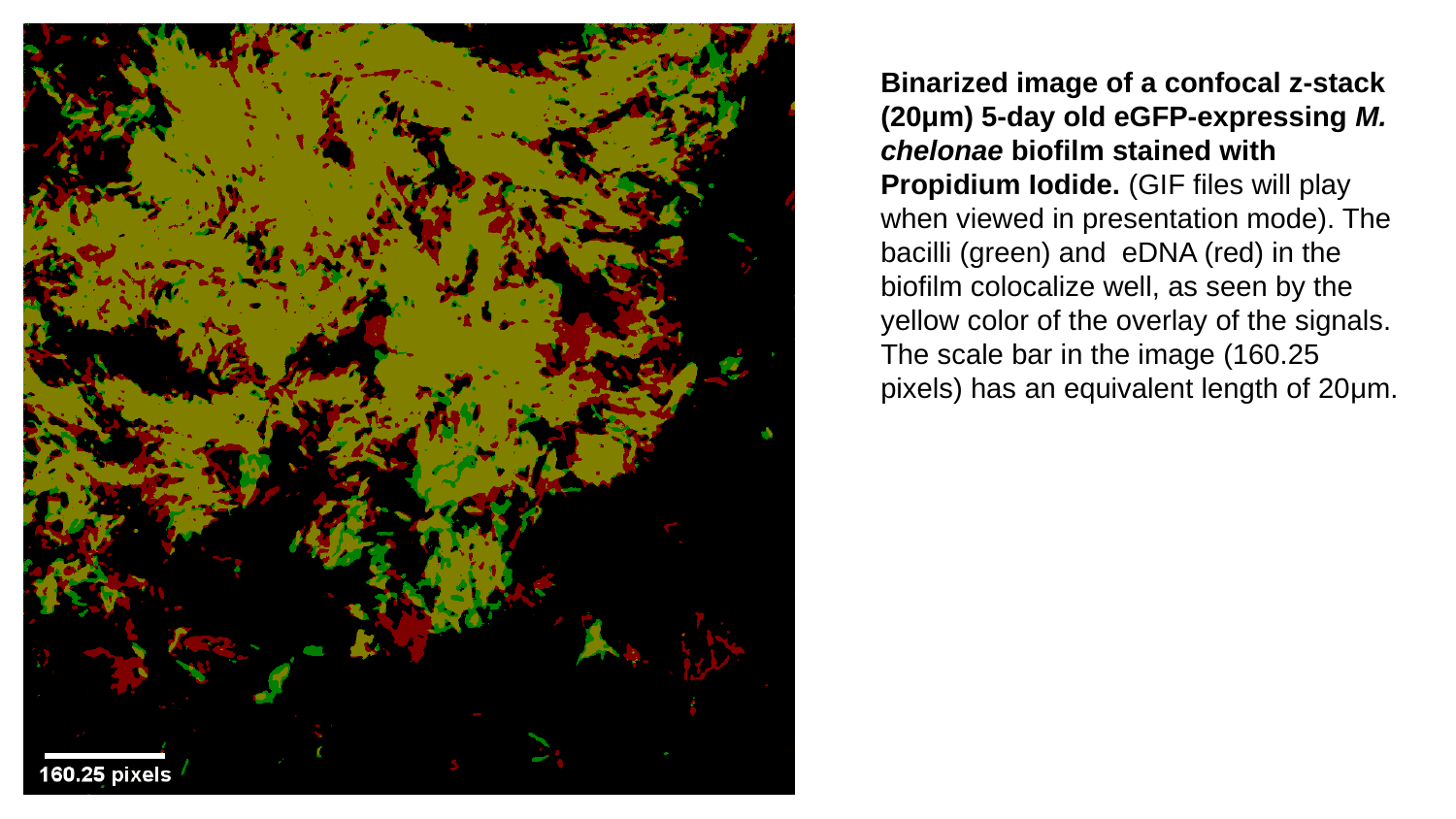

Binarized image of a confocal z-stack (20μm) 5-day old eGFP-expressing M. chelonae biofilm stained with Propidium Iodide. (GIF files will play when viewed in presentation mode). The bacilli (green) and eDNA (red) in the biofilm colocalize well, as seen by the yellow color of the overlay of the signals. The scale bar in the image (160.25 pixels) has an equivalent length of 20μm.

## Slide 3
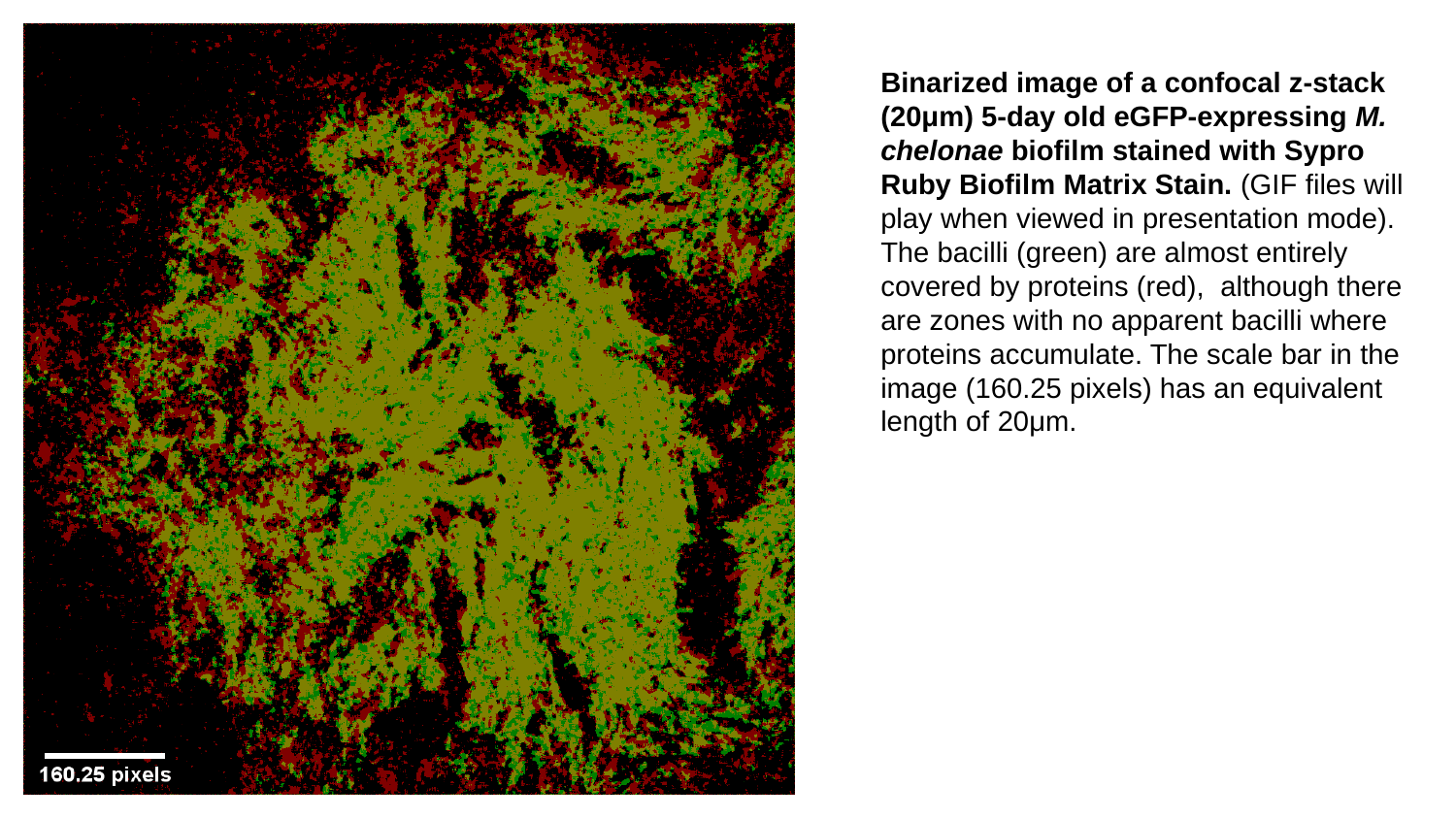

Binarized image of a confocal z-stack (20μm) 5-day old eGFP-expressing M. chelonae biofilm stained with Sypro Ruby Biofilm Matrix Stain. (GIF files will play when viewed in presentation mode). The bacilli (green) are almost entirely covered by proteins (red), although there are zones with no apparent bacilli where proteins accumulate. The scale bar in the image (160.25 pixels) has an equivalent length of 20μm.

## Slide 4
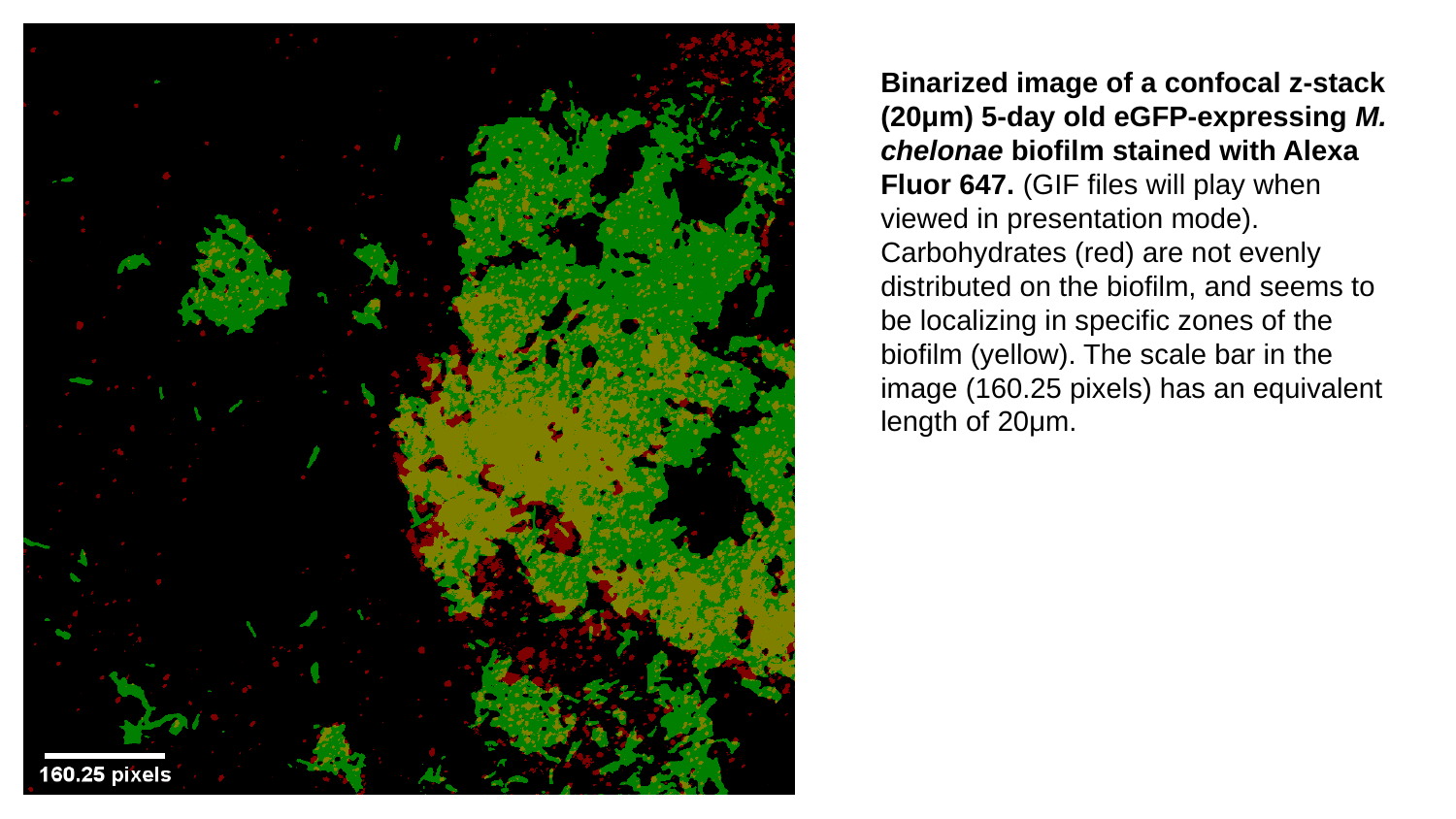

Binarized image of a confocal z-stack (20μm) 5-day old eGFP-expressing M. chelonae biofilm stained with Alexa Fluor 647. (GIF files will play when viewed in presentation mode). Carbohydrates (red) are not evenly distributed on the biofilm, and seems to be localizing in specific zones of the biofilm (yellow). The scale bar in the image (160.25 pixels) has an equivalent length of 20μm.
