# Supplementary material for: Biofilms of the non-tuberculous Mycobacterium chelonae form an extracellular matrix and display distinct expression patterns
Source: Cell Surf. 2020 Aug 5;6:100043. doi: 10.1016/j.tcsw.2020.100043 (PMC7421604; doi:10.1016/j.tcsw.2020.100043)
Supplement: Supplementary data 4 [file mmc4.docx]

**Enriched pathways during Biofilm t1 (Pellicle Early).**

| Subsystem | Fold change | Adjusted *P*-value | Number of genes | Genes |
| --- | --- | --- | --- | --- |
| L-α-aminoadipic acid biosynthesis | 3.92 | 0 | 1 | *BB28_RS18260* |
| Redox metabolism | 3.83 | 0.02 | 5 | *BB28_RS12850, BB28_RS12855, BB28_RS12860, BB28_RS12865, BB28_RS17655.* |
| Glyoxylate metabolism | 3.80 | 0.03 | 1 | *BB28_RS15360* |
| Lipid metabolism | 2.32 | 0.03 | 2 | *BB28_RS24120, BB28_RS24125.* |
| Mycolic acid biosynthesis | 6.30 | 0.02 | 3 | *BB28_RS03100, BB28_RS06770, BB28_RS06780.* |
| Membrane metabolism | -2.47 | 3.85E-06 | 5 | *BB28_RS10375, BB28_RS10385, BB28_RS10390, BB28_RS10395, BB28_RS11045* |
| Mycobactin biosynthesis | -2.50 | 3.33E-12 | 6 | *BB28_RS10375, BB28_RS10385, BB28_RS10390, BB28_RS10395, BB28_RS11045, BB28_RS11050.* |
| Transport | -3.32 | 4.43E-07 | 3 | *BB28_RS04470, BB28_RS04495, BB28_RS13025.* |

**Enriched pathways during Biofilm t2 (Pellicle Late).**

| Subsystem | Fold change | Adjusted *P*-value | Number of genes | Genes |
| --- | --- | --- | --- | --- |
| L-α-aminoadipic acid biosynthesis | 4.73 | 0 | 1 | *BB28_RS18260* |
| Arginine proline metabolism | -2.28 | 1.72E-06 | 5 | *BB28_RS11445, BB28_RS11450, BB28_RS11455, BB28_RS11460, BB28_RS11465.* |
| Mycobactin biosynthesis | -2.45 | 0.006 | 3 | *BB28_RS10385, BB28_RS10390, BB28_RS10395.* |
| Transport | -2.88 | 4.43E-07 | 15 | *BB28_RS04470, BB28_RS04490, BB28_RS04495, BB28_RS13025, BB28_RS20340, BB28_RS20920, BB28_RS20925, BB28_RS20930, BB28_RS20935, BB28_RS20940, BB28_RS20945, BB28_RS20950, BB28_RS20955, BB28_RS22830, BB28_RS23005.* |
